# Efficacy and safety of switching from entecavir to tenofovir alafenamide in chronic hepatitis B patients with low-level viremia: a real-world 48-week extension study

**DOI:** 10.1128/aac.01827-24

**Published:** 2025-02-04

**Authors:** Meng-Wen He, Li Cui, Dan-Dan Chen, Yun Zhao, Wen-Zhao Luo, Yun-Fei Jia, Jie Zhou, Qing-Juan He, Ying Dai, Wei-Hua Zhang, Zhao-Xia Yu, Wen-Chang Wang, Chang Guo, Yi-Ming Fu, Wu-Cai Yang, Xu-Yang Li, Yi-Fan Guo, Chun-Yan Wang, Jian-Jun Wang, Ping Li, Bing Qiao, Dong Ji, Zhong-Bin Li

**Affiliations:** 1 Peking University 302 Clinical Medical School 12465 , Beijing, China; 2 Senior Department of Hepatology, the Fifth Medical Center of PLA General Hospital 26460 , Beijing, China; 3 Department of Emergency, the Fifth Medical Center of PLA General Hospital 26460 , Beijing, China; 4 Department II of Infectious Diseases (Hepatology), The Second People’s Hospital of Jingzhou City, Jingzhou, China; 5 Department of Interventional Radiology, the Fifth Medical Center of PLA General Hospital 26460 , Beijing, China; 6 Department of Hepatobiliary & Gastrointestinal Diseases, Henan Province Hospital of Traditional Chinese Medicine 658459 , Zhengzhou, Henan, China; 7 Department of Infectious Diseases, Xinjiang Uygur Autonomous Region Infectious Disease Hospital, Xinjiang, China; 8 Department II of Gastroenterology, The Eighth People’s Hospital of Qingdao, Qingdao, China; 9 Department of Gastroenterology, Qiqihar Seventh Hospital, Qiqihar, China; 10 The Second ward, Qian 'an Infectious Disease Hospital, Tangshan, China; 11 Liver Oncology Department, The Sixth People’s Hospital of Qingdao, Qingdao, China; 12 Chinese PLA Medical School 104607 , Beijing, Beijing, China; 13 Integrated TCM & Western Medicine Department, Tianjin Second People’s Hospital, Tianjin, China; University of Pittsburgh School of Medicine, Pittsburgh, Pennsylvania, USA

**Keywords:** low-level viremia, chronic hepatitis B, complete virological response, ALT normalization, lipid effect, renal safety

## Abstract

Chronic hepatitis B (CHB) patients receiving entecavir (ETV) treatment might develop low-level viremia (LLV), which is proven to be associated with worse clinical outcomes, such as risk of drug-related mutations, progression to cirrhosis, and even hepatocellular carcinoma. This real-world prospective study evaluated the efficacy and safety of switching from ETV to tenofovir alafenamide fumarate (TAF) in CHB patients with LLV. From August 2020 to August 2023, 351 ETV-experienced CHB patients with LLV were enrolled from eight hospitals. Patients either continued ETV or switched to TAF. The primary efficacy endpoint was the complete virological response (CVR) at week 48; the safety endpoint was the first occurrence of any clinical adverse event during the treatment; and the renal safety and change in blood lipids were also assessed. Inverse probability treatment weighting (IPTW) generated 350.9 cases in the ETV group and 351.4 cases in the TAF group. After the 48-week treatment, the CVR and ALT normalization rates in the TAF group were 75.3% and 67.8%, which were significantly higher than 11.4% and 17.1% in the ETV group (*P* < 0.001). The two strategies showed comparable impact on renal function and lipid profiles, regarding low-density lipoprotein (LDL) cholesterol and the total cholesterol to high-density lipoprotein (TC/HDL) ratio. Therefore, for ETV-treated patients with LLV, switching to TAF is superior compared with continuing ETV treatment in terms of virological and biochemical response, with non-inferior renal safety and lipid profiles.

**CLINICAL TRIALS:**

This study is registered with the Chinese Clinial Trial Registry as ChiCTR2400089257.

## INTRODUCTION

Chronic hepatitis B (CHB) infection is the most common chronic viral infection worldwide, affecting approximately 257 million people carrying hepatitis B virus (HBV) (1). Nucleos(t)ide analogs (NAs) are effective in suppressing HBV replication significantly and have a favorable safety profile. Entecavir (ETV), tenofovir disoproxil fumarate (TDF), and tenofovir alafenamide fumarate (TAF) are the first-line NAs for CHB patients due to its high antiviral potency and genetic barrier to resistance (2). However, despite most patients could achieve complete virological response (CVR) with ETV treatment at week 48, approximately 10%–40% of patients experience persistent or intermittent low-level viraemia (LLV, HBV DNA between 20 and 2,000 IU/mL) (3, 4).

Studies have shown that patients with liver fibrosis progression after 78 weeks of antiviral treatment had a higher detection rate of HBV DNA (50%) compared with those with liver fibrosis regression (19%) or indetermination (26%), suggesting that LLV may promote liver fibrosis progression (5). Moreover, low positive values of HBV DNA had been associated with disease progression and an increased risk of hepatocellular carcinoma (HCC) despite receiving highly potent antiviral treatment (6, 7). Failure to achieve a CVR has been identified as a significant risk factor for HCC in patients with cirrhosis treated with ETV (8).

Thus, developing a more reasonable rescue treatment strategy for patients with LLV is necessary. For such patients, a long-term benefit can be achieved with efficient rescue antiviral therapy. Tenofovir alafenamide fumarate (TAF) is an orally bioavailable prodrug of tenofovir (TFV), which inhibits the reverse transcription of HBV DNA. TAF has great plasma stability allowing delivery of the active metabolite to hepatocytes efficiently at a dose of 25 mg with low circulating levels of TFV, reducing the risk of renal and bone injury with long-term use (9, 10). However, studies have shown TAF was inferior to TDF in terms of fasting lipid change, with the precise mechanism for these changes not being ascertained (11).

To optimize existing rescue treatment strategies until new drugs are developed to cure HBV, several of the following questions remain: Should Patients with LLV choose to switch to another first-line agent or add a second drug or continue the original treatment, or combine with interferon? These problems need to be further studied and solved to understand the relationship between HBV DNA replication and clearance. The treatment strategy of patients with LLV remains a noteworthy issue. A previous 24-week single-center study by our group suggested the potential benefit of switching to TAF in patients with LLV on ETV (4). However, the long-term efficacy and safety of this strategy, particularly in a broader patient population, remained unclear. Therefore, this prospective, multi-center study aimed to evaluate the 48-week efficacy and safety of switching to TAF versus continuing ETV in CHB patients with LLV on ETV, providing crucial insights into long-term outcomes and generalizability.

## MATERIALS AND METHODS

### Study population

This multicenter, prospective, 48-week extension study extended the follow-up of a previously reported 24-week cohort. Patients were recruited from eight participating hospitals between August 2020 and August 2023. We enrolled patients who met all of the following inclusion criteria: (i) aged over 18 years; (ii) serum positive for both HBsAg and HBV DNA (12); and (iii) received ETV treatment for >48 weeks and experienced LLV (12). Among them, we excluded patients who met one of the following exclusion criteria: (i) treated with other NAs or interferon; (ii) poor clinical compliance; (iii) with virological resistance to ETV; (iv) coinfected with either hepatitis C virus or human immunodeficiency virus; (v) combined with another liver disease including autoimmune liver disease (13) and drug-induced liver injury (14) etc.,; (vi) had decompensated cirrhosis (eg, complicated with obvious ascites, variceal hemorrhage, or encephalopathy); (vii) any cancers, and (viii) women with pregnancy or breastfeeding. This study was registered at the Chinese Clinical Trial Registry (ChiCTR2400089257).

**Fig 1:**
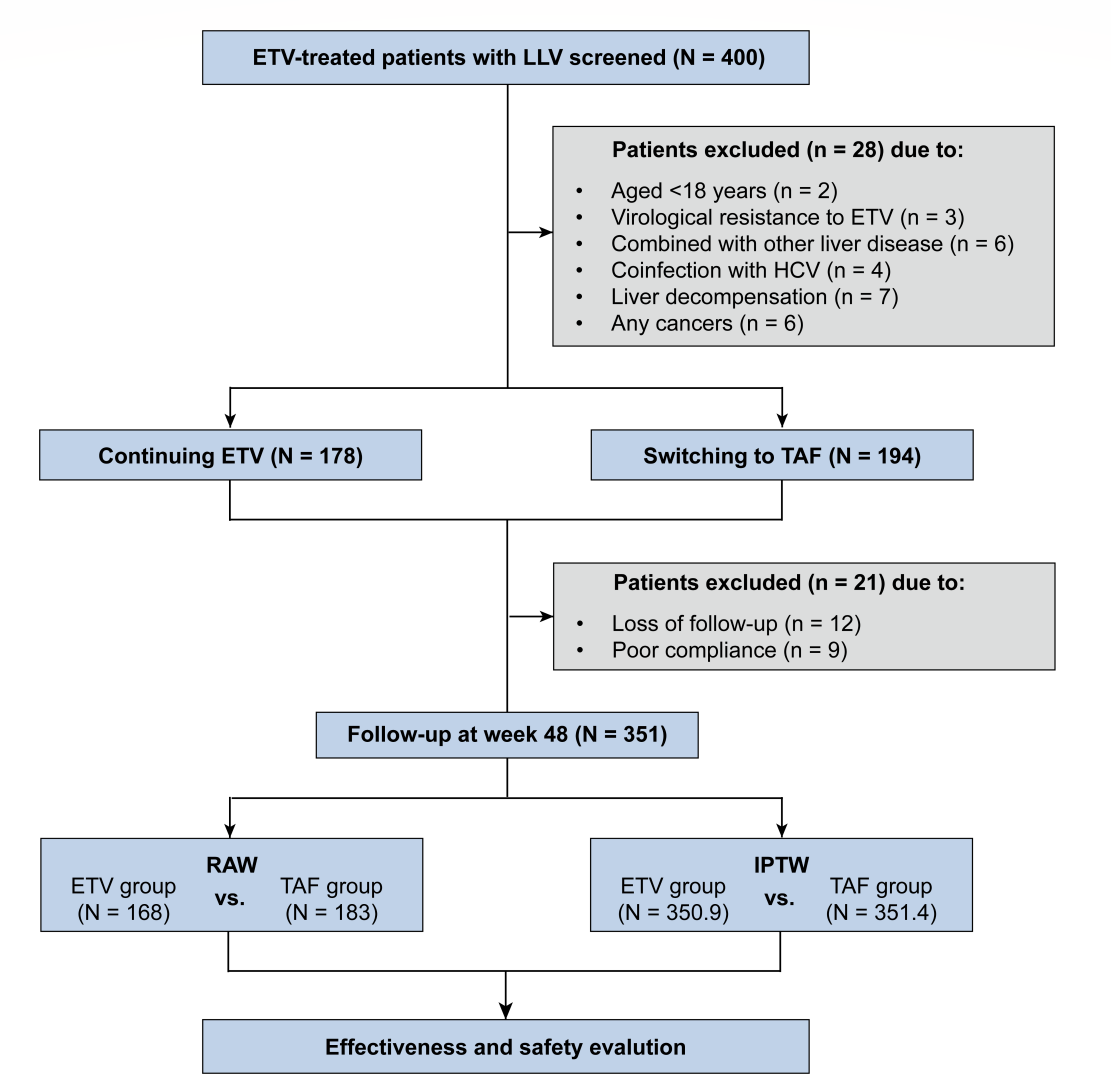
Flow chart of the study population. LLV, low-level viremia (HBV DNA >20 IU/mL and <2,000 IU/mL); IPTW, inverse probability treatment weighting; ETV, entecavir; TAF, tenofovir alafenamide fumarate.

All the enrolled patients received either continuing ETV (0.5 mg/d) or switching to TAF (25 mg/d) therapy based on the patient’s discretion after the cost, efficacy, the safety of both therapies, and long-term outcomes of LLV had been explained. A flow chart of the study design is presented in Fig. 1.

The study was approved by the ethics committees of all participating institutions and conducted in compliance with the 1975 Declaration of Helsinki, Good Clinical Practice guidelines, and local regulatory requirements.

### Measurements

Patients were assessed by physical examinations, blood tests (virological, biochemical parameters), and liver stiffness measurements (LSM) at baseline, week 12, week 24, week 36, and week 48, respectively. Serum HBV DNA levels were measured by COBAS TaqMan HBV Test (Roche, USA), with 20 IU/mL as the lower limit of detection. Serum quantitative hepatitis B surface antigen (qHBsAg) was measured by chemiluminescent immunoassay using the HBsAg QT assay kit (Roche, USA). HBV serological markers were measured by chemiluminescent immunoassay (Abbott, Germany). The HBV genotypes were identified by the PCR fluorescent probe method. For resistance testing, the HBV reverse transcriptase gene was amplified by nested PCR and then detected by direct population sequencing. The estimated glomerular filtration rate (eGFR) was determined by the Cockcroft-Gault method. Cirrhosis was diagnosed with ultrasound. LSM was performed by experienced operators using transient elastography (FibroTouch; HISKY, China). APRI and FIB-4 scores were calculated for all patients based on the following formulas: APRI = ([AST/ULN]/PLT) × 100 and FIB-4 = (age ×AST)/(PLT×ALT^0.5^), (AST ULN = 40 U/L). Clinical compliance was assessed with 8-item Morisky medication adherence scale (MMAS-8). At the treatment baseline, any patient should be determined whether there was a disease such as diabetes mellitus or hyperlipidemia. Drinking was defined as alcohol intake of 25–40 g/day for men and 15–20 g/day for women lasting at least 5 years (15).

### Outcomes

The primary efficacy endpoint was complete virological response (CVR) defined as serum HBV DNA level <20 IU/mL at week 48. Secondary efficacy endpoints were the degree of serum HBV DNA levels decreased in patients with detectable HBV DNA; ALT normalization (i.e., ALT ≤ULN at week 48 in the subset with ALT >ULN at baseline); degree of qHBsAg decline; HBeAg loss and seroconversion; and changes in liver fibrosis as measured by LSM, APRI, and FIB-4.

The primary safety endpoint was the first occurrence of any clinical adverse event or drug discontinuation. The secondary safety endpoints were the changes in renal function, as determined by serum creatinine, eGFR, phosphorus, urinary β2-microglobulin (Uβ2-MG), urinary N-acetyl-β-D-glucosaminidase (UNAG), and urine microalbumin, and the changes in fasting lipid parameters, as determined by total cholesterol (TC), triglycerides (TG), high-density lipoprotein (HDL) cholesterol, low-density lipoprotein (LDL) cholesterol, and TC/HDL ratio. All adverse events (AEs), except virologic response, were graded according to the Common Terminology Criteria for Adverse Events (version 5.0) [CTCAE version 5.0] (16). All patients fulfilled 8-item Morisky medication adherence sca1e(MMAS-8）regarding compliance at each visit.

### Statistical analysis

Categorical variables were expressed as numbers (percentages) and compared using a chi-squared or Fisher’s exact test. Continuous variables with normal distribution were expressed as mean ± standard deviation (SD) and compared with the student’s *t*-test, continuous variables with skewed distribution were expressed as median (interquartile range, IQR) and compared with the Mann-Whitney test. The 95% confidence intervals (CI) were calculated for each predictive test.

Inverse probability treatment weighting (IPTW) was used to reduce the effect of selection bias and potential confounding variables by observed baseline characteristics. A logistic regression model was established to calculate the propensity score, which included the following baseline covariates: serum HBeAg status, cirrhosis, drink, and LSM. Using this method, the weights for patients in the TAF group were set to the inverse of the propensity score, and for those patients in the ETV group, the weights were set to the inverse of (1: propensity score). Therefore, the average treatment effect (ATE) was obtained after applying IPTW.

A *P* value < 0.05 was considered significant for all statistical tests. All of the statistical analyses were performed using R software (version 4.1.3).

## RESULTS

### Baseline characteristics

In total, 400 patients were screened, and 351 patients were enrolled according to including and excluding criteria. One hundred sixty-eight (47.9%) patients received continuing ETV monotherapy (ETV group), and 183 (52.1%) patients received switching to TAF treatment (TAF group). The percent of male patients was 82.1% (288/351), and the median age was 47 years; the median HBV DNA level and ALT at baseline was 246 IU/ mL and 31 U/L. The median serum qHBsAg was 3521 IU/mL. Serum HBeAg was positive in 61.5% (216/351) of patients. The median LSM, APRI, and FIB-4 were 7.2 kPa, 0.48, and 1.51, respectively, and 32.5% (114/351) patients were diagnosed with cirrhosis.

To address potential confounding, we conducted an IPTW analysis to balance the baseline covariates between the ETV and TAF groups, thereby providing a more robust estimate of the treatment effect. The details of baseline characteristics in the RAW and IPTW cohorts are shown in Table 1.

**TABLE 1:** Baseline clinical characteristics of enrolled patients^*a*^

	IPTW (*N* = 702.4)					RAW (*N* = 351)		
	Overall (*N* = 702.3)	ETV (*N* = 350.9)	TAF (*N* = 351.4)	*P* value	Overall (*N* = 351)	ETV (*N* = 168)	TAF (*N* = 183)	*P* value
Male sex, n (%)	575.3 (81.9)	277.8 (79.2)	297.5 (84.7)	0.197	288 (82.1)	134 (79.8)	154 (84.2)	0.352
Age, years	47 (39–56)	47 (40–56)	47 (38–55)	0.989	47 (39–55)	47 (40–54)	47 (39–56)	0.468
BMI, kg/m^2^	21.40 (19.58–24.00)	21.50 (19.50–23.96)	21.40 (19.60–24.00)	0.654	21.50 (19.57–24.00)	21.55 (19.50–24.00)	21.40 (19.60–24.05)	0.636
qHBsAg, IU/mL	3548 (534–11859)	3889 (626–11879)	3334 (461–11577)	0.768	3521 (548–11859)	3889 (634–18206)	2882 (460–10241)	0.240
HBV DNA, IU/mL	253 (98–824)	232 (98–747)	295 (98–890)	0.299	246 (99–818)	231 (103–699)	290 (97–879)	0.361
HBeAg positive, n (%)	436.1 (62.1)	218.1 (62.2)	218.0 (62.0)	0.980	216 (61.5)	114 (67.9)	102 (55.7)	0.026
ALT, U/L	31 (22–48)	30 (22–46)	31 (22–49)	0.642	31 (22–48)	31 (22–49)	31 (21–47)	0.903
AST, U/L	31 (22–42)	32 (22–42)	30 (22–45)	0.894	31 (22–42)	33 (22–42)	29 (22–45)	0.741
TBIL, µmol/L	13.7 (10.0–17.7)	13.3 (10.0–17.7)	13.8 (10.1–17.6)	0.647	13.7 (10.0–17.7)	13.3 (10.0–17.6)	13.9 (10.2–18.0)	0.466
PLT, ×10^9^ /L	173 (121–214)	170 (112–210)	175 (127–217)	0.295	170 (119–213)	170 (112–210)	173 (123–215)	0.496
Creatinine, µmol/L	75.0 (65.0–84.7)	75.0 (66.0–84.0)	74.2 (63.0–86.0)	0.705	75.0 (64.0–84.0)	75.0 (66.0–84.0)	74.0 (62.6–85.5)	0.471
eGFR, mL/min/1.73 m^2^	100.7 (90.0–111.2)	100.0 (90.0–110.0)	102.0 (90.0–112.2)	0.783	100.7 (90.0–111.0)	100.0 (90.0–110.0)	102.0 (90.5–112.5)	0.607
Phosphorus, mmol/L	1.16 (1.02–1.31)	1.17 (1.04–1.29)	1.15 (1.00–1.32)	0.788	1.16 (1.02–1.31)	1.17 (1.03–1.29)	1.15 (1.00–1.32)	0.838
Uβ2-MG, mg/L	0.17 (0.11–0.29)	0.17 (0.12–0.29)	0.16 (0.09–0.28)	0.278	0.16 (0.11–0.28)	0.17 (0.12–0.28)	0.16 (0.10–0.28)	0.396
UNAG, U/L	10.3 (6.5–16.9)	10.8 (6.5–18.8)	9.9 (6.4–15.3)	0.366	10.2 (6.5–16.9)	11.3 (6.5–19.4)	9.8 (6.4–15.0)	0.159
urine microalbumin, mg/L	7.0 (3.0–11.0)	7.0 (4.0–11.0)	6.0 (3.0–13.0)	0.985	7.0 (3.0–11.5)	7.0 (4.0–11.0)	6.0 (3.0–12.5)	0.837
TC, mmol/L	3.40 (2.80–4.09)	3.50 (2.82–4.22)	3.30 (2.71–3.97)	0.154	3.38 (2.80–4.10)	3.49 (2.80–4.22)	3.30 (2.72–3.99)	0.251
TG, mmol/L	0.87 (0.62–1.22)	0.86 (0.63–1.17)	0.88 (0.61–1.24)	0.800	0.88 (0.62–1.23)	0.86 (0.63–1.21)	0.88 (0.62–1.26)	0.621
HDL, mmol/L	0.99 (0.80–1.22)	0.99 (0.77–1.24)	0.99 (0.82–1.18)	0.778	0.99 (0.80–1.22)	0.99 (0.77–1.23)	0.99 (0.82–1.18)	0.883
LDL, mmol/L	2.20 (1.80–2.73)	2.29 (1.80–2.75)	2.20 (1.77–2.70)	0.406	2.20 (1.79–2.73)	2.28 (1.80–2.74)	2.20 (1.79–2.71)	0.584
TC/HDL ratio	3.37 (2.78–4.32)	3.51 (2.80–4.37)	3.29 (2.74–4.27)	0.232	3.35 (2.78–4.32)	3.47 (2.79–4.36)	3.29 (2.75–4.27)	0.377
Child-Pugh B class, n (%)	121.8 (17.3)	64.9 (18.5)	56.9 (16.2)	0.582	61 (17.4)	31 (18.5)	30 (16.4)	0.713
Cirrhosis, n (%)	227.8 (32.4)	114.1 (32.5)	113.7 (32.3)	0.973	114 (32.5)	44 (26.2)	70 (38.3)	0.022
LSM, kPa	7.2 (6.5–9.5)	7.4 (6.5–9.8)	7.1 (6.4–9.5)	0.579	7.2 (6.5–9.6)	7.5 (6.6–9.9)	7.0 (6.2–9.5)	0.105
APRI	0.48 (0.30–0.79)	0.50 (0.31–0.76)	0.45 (0.28–0.79)	0.425	0.48 (0.30–0.79)	0.50 (0.32–0.76)	0.46 (0.29–0.80)	0.437
FIB-4	1.52 (0.98–2.54)	1.56 (1.06–2.53)	1.39 (0.94–2.54)	0.357	1.51 (1.00–2.51)	1.55 (1.06–2.43)	1.48 (0.96–2.53)	0.610
Diabetes mellitus, n (%)	121.3 (17.3)	59.9 (17.1)	61.4 (17.5)	0.923	57 (16.2)	28 (16.7)	29 (15.8)	0.950
Drinker, n (%)	178.4 (25.4)	88.5 (25.2)	89.8 (25.6)	0.945	90 (25.6)	53 (31.5)	37 (20.2)	0.021
Family history, n (%)	388.0 (55.2)	190.3 (54.2)	197.7 (56.3)	0.714	196 (55.8)	92 (54.8)	104 (56.8)	0.778

^*a*^ BMI, body mass index; qHBsAg, quantitative hepatitis B surface antigen; HBV, hepatitis B virus; HBeAg, hepatitis B e antigen; ALT, alanine aminotransferase; AST, aspartate aminotransferase; TBIL, total bilirubin; PLT, platelet; eGFR, estimated glomerular filtration rate; Uβ2-MG, urinary β2-microglobulin; UNAG, urinary N-acetyl-β-D-glucosaminidase; TC, total cholesterol; TG, triglyceride; HDL, high-density lipoprotein; LDL, low-density lipoprotein; LSM, liver stiffness measurement; APRI, aspartate aminotransferase-to-platelet ratio index; FIB-4, fibrosis index based on four factors; DM, diabetes mellitus.

### Virological response

**Fig 2:**
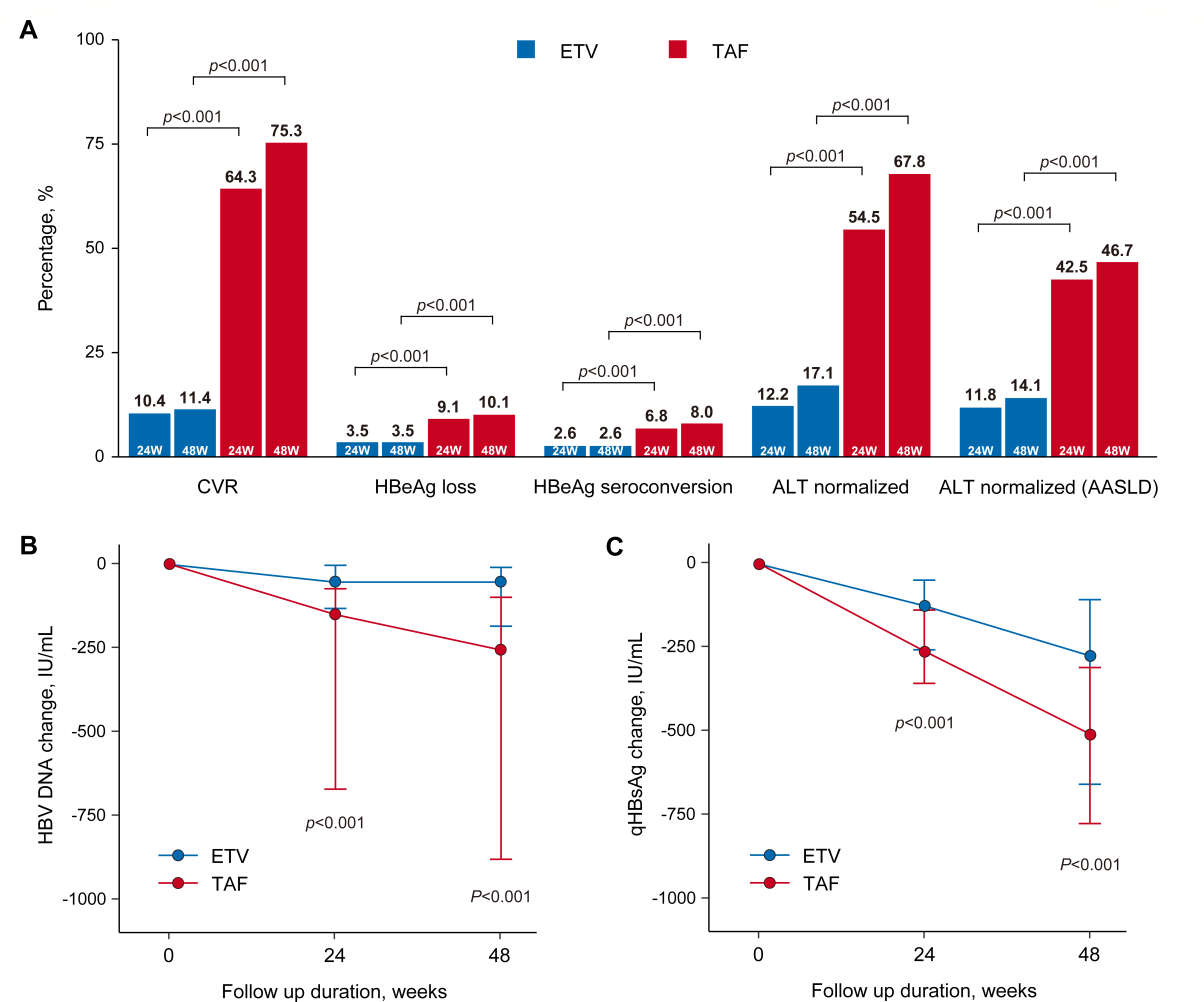
Virological and biochemical responses over the 48  weeks. (**A**) Virological and biochemical responses at week 48, including CVR, HBeAg loss and seroconversion, and ALT normalization. ALT normalization is presented based on both central laboratory criteria (left) and the AASLD guidelines (right). (**B**) Change in HBV DNA from baseline through week 48. (**C**) Change in qHBsAg from baseline through week 48. Bars in panels B and C represent the median change from baseline, with the first and third quartiles indicated. HBeAg loss and seroconversion were assessed only in patients who were seropositive for HBeAg and seronegative for anti-HBe at baseline. CVR, complete virological response; HBeAg, hepatitis B e antigen; ALT, alanine aminotransferase; AASLD, American Association for the Study of Liver Diseases; HBV, hepatitis B virus; qHBsAg, quantitative hepatitis B surface antigen; TAF, tenofovir alafenamide; ETV, entecavir.

In the IPTW cohort, the CVR rate was significantly higher in the TAF group (64.3%) compared with the ETV group (10.4%) at week 24. This difference was also statistically significant at week 48 (75.3% vs. 11.4%, *P* < 0.001; Fig. 2A). Furthermore, the median HBV DNA decline was significantly greater in the TAF group compared with the ETV group at both week 24 (150 IU/mL vs 53 IU/ml, *P* < 0.001) and week 48 (255 IU/mL vs 53 IU/ml, *P* < 0.001; Fig. 2B). Similar findings were observed in the raw cohort (Table 2).

**TABLE 2:** Efficacy assessment^*a*^

IPTW	Week 24			Week 48		
	ETV (*N* = 350.9)	TAF (*N* = 351.4)	*P* value	ETV (*N* = 350.9)	TAF (*N* = 351.4)	*P* value
CVR, n (%)	36.6 (10.4)	226.0 (64.3)	<0.001	40.1 (11.4)	264.8 (75.3)	<0.001
HBV DNA decrease, IU/mL	53 (3–131)	150 (73–670)	<0.001	53 (9–184)	255 (98–879)	<0.001
qHBsAg decrease, IU/mL	125 (49–256)	261 (138–356)	<0.001	274 (107–657)	508 (309–774)	<0.001
HBeAg loss, n/N (%)^*b*^	7.7/217.4 (3.5)	19.6/216.1 (9.1)	<0.001	7.7/217.4 (3.5)	21.9/216.1 (10.1)	<0.001
HBeAg seroconversion, n/N (%)^*b*^	5.7/217.4 (2.6)	14.8/216.1 (6.8)	<0.001	5.7/217.4 (2.6)	17.2/216.1 (8.0)	<0.001
Normalized ALT, n/N (%)^*c*^	13.3/109.2 (12.2)	68.1/124.9 (54.5)	<0.001	18.7/109.2 (17.1)	84.7/124.9 (67.8)	<0.001
Normalized ALT, n/N (%)^*d*^	19.3/163.2 (11.8)	66.9/157.5 (42.5)	<0.001	23.0/163.2 (14.1)	73.6/157.5 (46.7)	<0.001
LSM decrease, kPa	0.1 (-0.2–0.3)	0.1 (-0.3–0.3)	0.221	0.1 (-0.2–0.4)	0.1 (-0.2–0.5)	0.928
APRI decrease	0.03 (-0.04–0.10)	0.03 (-0.02–0.13)	0.354	0.01 (-0.02–0.04)	0.01 (-0.01–0.05)	0.354
FIB-4 decrease	−0.01 (-0.25–0.19)	0.00 (-0.15–0.25)	0.188	0.04 (-0.23–0.34)	0.00 (-0.20–0.38)	0.757

RAW	Week 24			Week 48		
	ETV (*N* = 168)	TAF (*N* = 183)	*P* value	ETV (*N* = 168)	TAF (*N* = 183)	*P* value
CVR, n (%)	18 (10.7)	123 (67.2)	<0.001	20 (11.9)	142 (77.6)	<0.001
HBV DNA decrease, log_10_ IU/mL	52 (2–133)	149 (70–649)	<0.001	55 (9–180)	270 (97–874)	<0.001
qHBsAg decrease, log_10_ IU/mL	131 (50–265)	262 (139–356)	<0.001	273 (103–662)	531 (322–747)	<0.001
HBeAg loss, n/N (%)^*b*^	4/114 (3.5)	10/102 (9.8)	<0.001	4/114 (3.5)	11/102 (9.8)	<0.001
HBeAg seroconversion, n/N (%)^*b*^	3/114 (2.6)	7/102 (6.9)	<0.001	3/114 (2.6)	8/102 (7.8)	<0.001
Normalized ALT, n/N (%)^*c*^	6/53 (13.2)	35/64 (54.7)	<0.001	9/53 (17.0)	43/64 (67.2)	<0.001
Normalized ALT, n/N (%)^*d*^	9/80 (11.3)	33/80 (41.3)	<0.001	12/80 (15.0)	37/80 (46.3)	<0.001
LSM decrease, kPa	0.1 (-0.2–0.4)	0.1 (-0.3–0.3)	0.058	0.1 (-0.2–0.4)	0.1 (-0.2–0.4)	0.673
APRI decrease	0.03 (-0.04–0.10)	0.03 (-0.02–0.15)	0.200	0.01 (-0.02–0.04)	0.01 (-0.01–0.06)	0.200
FIB-4 decrease	−0.02 (-0.24–0.19)	0.00 (-0.15–0.24)	0.144	0.04 (-0.22–0.34)	0 (-0.20–0.36)	0.981

^*a*^ CVR, complete virological response; qHBsAg, quantitative hepatitis B surface antigen; HBV, hepatitis B virus; HBeAg, hepatitis B e antigen; ALT, alanine aminotransferase; AASLD, American Association for the Study of Liver Diseases; LSM, liver stiffness measurement; APRI, aspartate aminotransferase-to-platelet ratio index; FIB-4, fibrosis index based on four factors; TAF, tenofovir alafenamide; ETV, entecavir.

^*b*^ Among patients who were seropositive for HBeAg and negative for anti-HBe at baseline.

^*c*^ Among patients with ALT at baseline above the central lab criteria (≤ 40 U/L).

^*d*^ Among patients with ALT at baseline above AASLD defined criteria (≤ 35 U/L for males and ≤25 U/L for females).

### ALT normalization

In the IPTW cohort, at week 48, ALT normalization in the TAF group was significantly higher than that in the ETV group. Under our hospital central laboratory criteria (≤40 U/L), it was 67.8% in the TAF group and 17.1% in the ETV group (*P* < 0.001); under American Association for the Study of Liver Diseases (AASLD) criteria (≤35 U/L for males and ≤25 U/L for females), it was 46.7% in TAF group or 14.1% in ETV group (*P* < 0.001). At week 24, the ALT normalization in two cohorts also showed statistically significant differences (*P* < 0.001; Fig. 2A). In raw analysis at week 48, the results were similar (Table 2).

### HBV biomarker change

In the IPTW cohort, analysis revealed no patients achieved complete serum HBsAg clearance during the 48-week treatment period. However, the TAF group consistently showed a significantly greater decline in median serum qHBsAg compared with the ETV group. This difference was statistically significant at both weeks 24 (261 IU/mL vs. 125 IU/ml, *P* < 0.001) and 48 (508 IU/mL vs. 274 IU/ml, *P* < 0.001; Fig. 2C). Furthermore, TAF treatment was associated with significantly higher rates of both HBeAg loss (10.1% vs. 3.5%, *P* < 0.001) and seroconversion (8.0% vs. 2.6%, *P* < 0.001) at week 48 compared with ETV (Fig. 2C). These findings were corroborated by RAW analysis at week 48 (Table 2).

### Non-invasive liver fibrosis measurements

No significant changes were observed in LSM, APRI, or FIB-4 scores in either the ETV or TAF groups from baseline to week 48. In the ETV group, median LSM values were 7.4 kPa at baseline and 7.1 kPa at week 48 (*P* = 0.391), whereas median APRI scores were 0.50 and 0.48 (*P* = 0.741), and median FIB-4 scores were 1.56 and 1.49 (*P* = 0.731), respectively. Similarly, the TAF group showed median LSM values of 7.1 kPa at both baseline and week 48 (*P* = 0.674), with median APRI scores of 0.45 and 0.44 (*P* = 0.614), and median FIB-4 scores of 1.39 and 1.42 (*P* = 0.733). Furthermore, no significant differences were found between the ETV and TAF groups in the changes for LSM (*P* = 0.221 at week 24, *P* = 0.928 at week 48), APRI (*P* = 0.354 at week 24, *P* = 0.354 at week 48), or FIB-4 (*P* = 0.188 at week 24, *P* = 0.757 at week 48; Table 2).

### BMI and fasting lipid change

**Fig 3:**
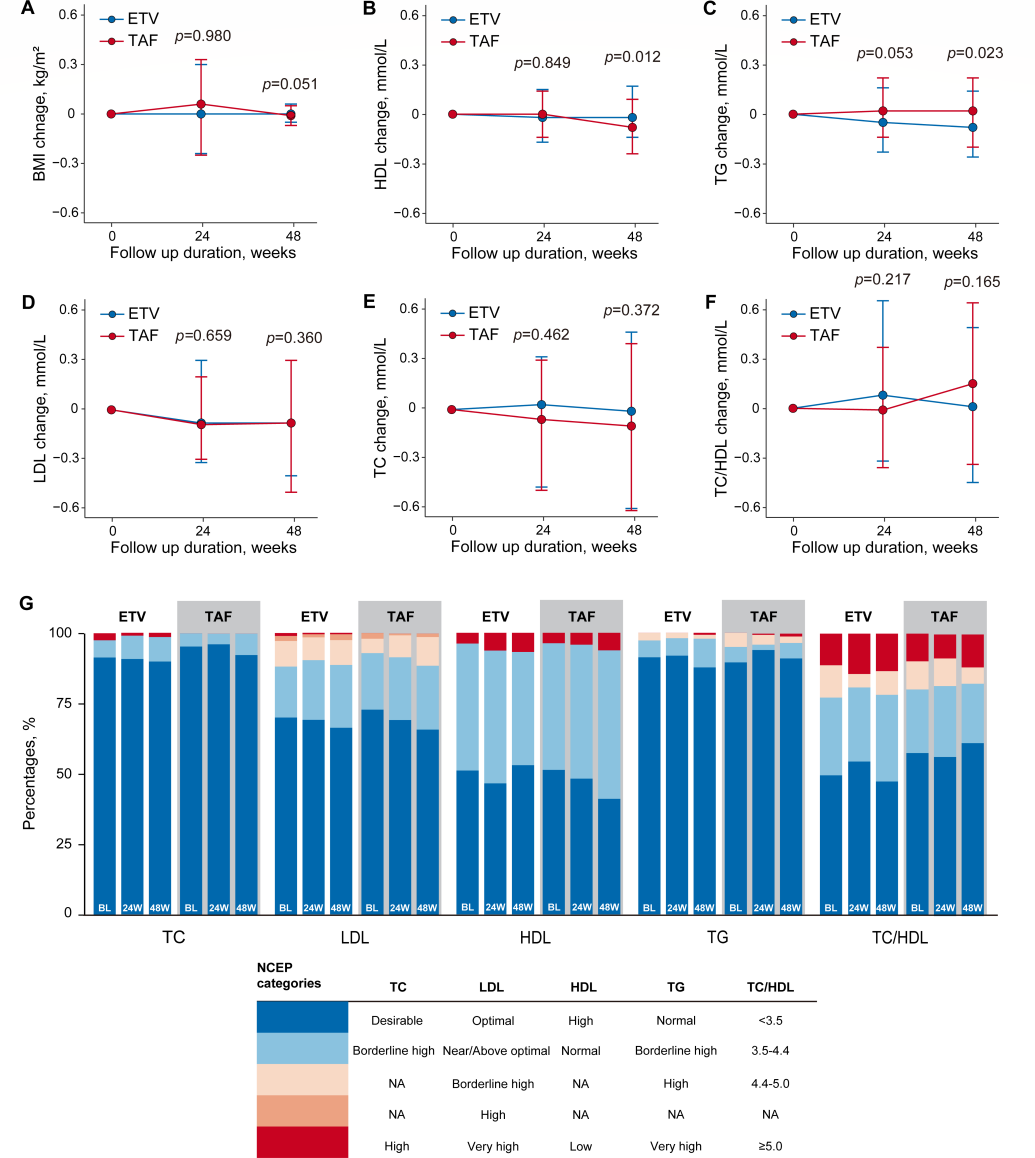
BMI and fasting lipids change over the 48  weeks. (**A**) Change in BMI and fasting lipids from baseline through week 48. (**B to G**) Change from baseline in fasting lipids at week 48 by National Cholesterol Education Program (NCEP) category. Bars are expressed as median change from baseline (first–third quartile). BMI, body mass index; TC, total cholesterol; LDL, low-density lipoprotein; HDL, high-density lipoprotein; TG, triglyceride; TAF, tenofovir alafenamide; ETV, entecavir.

Neither the ETV nor the TAF treatment groups showed significant changes in BMI at week 24 (*P* = 0.980) and week 48 (*P* = 0.051), indicating that both treatments had minimal impact on BMI (Fig. 3A).

Changes from baseline in HDL levels (Fig. 3B) and TG levels (Fig. 3C) favored ETV-based regimens; however, neither group exhibited significant changes from baseline (all *P* value < 0.05). Additionally, there were no differences observed between the groups in LDL (Fig. 3D), TC (Fig. 3E), and TC/HDL ratio changes (Fig. 3F). In the week 24 and 48 analyses of lipids based on National Cholesterol Education Program categories, there were no significant differences in the changes in lipid profiles between the groups, and the TAF group was not inferior to the ETV group (Fig. 3G).

### Renal function changes

**Fig 4:**
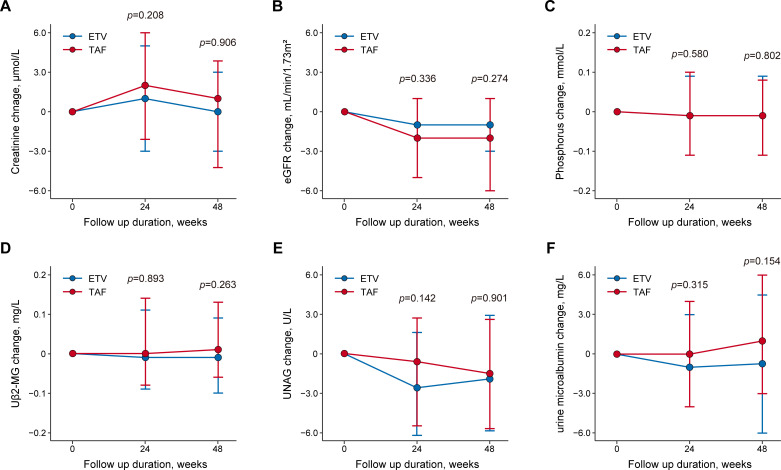
Changes from baseline in (**A**) creatinine, (**B**) eGFR, (**C**) phosphorus, (**D**) Uβ2-MG, (**E**) UNAG, and (**F**) urine microalbumin over 48 weeks. Bars are expressed as median change from baseline (first-third quartile). eGFR, estimated glomerular filtration rate; Uβ2-MG, urinary β2-microglobulin; UNAG, urinary N-acetyl-β-D-glucosaminidase.

Creatinine levels and eGFR exhibited similar trends in both treatment groups, with no significant changes observed from baseline to weeks 24 and 48. Specifically, the change in creatinine levels from baseline to weeks 24 and 48 was not significantly different between the ETV and TAF groups (week 24: 1.0 µmol/L vs. 2.0 µmol/L, *P* = 0.208; week 48: 0 µmol/L vs. 1 µmol/L, *P* = 0.906, Fig. 4A). Similarly, eGFR showed no significant between-group differences in the change from baseline to weeks 24 and 48 (week 24: −1.0 mL/min/1.73 m^2^ vs. −2.0 mL/min/1.73 m^2^, *P* = 0.336; week 48: −1.0 mL/min/1.73 m^2^ vs. −2.0 mL/min/1.73 m^2^, *P* = 0.274, Fig. 4B). The phosphorus (Fig. 4C), Uβ2-MG (Fig. 4D), UNAG (Fig. 4E), and urine microalbumin (Fig. 4F) levels also showed no significant differences in changes from baseline to weeks 24 and 48 between the ETV and TAF groups. No patient in either group experienced a renal serious adverse event due to study drugs, an event of proximal tubulopathy (including Fanconi syndrome), or an event of renal failure (Table 3).

**TABLE 3:** Safety data at week 48

	ETV (*N* = 168)	TAF (*N* = 183)
Patients with any adverse event, n (%)	58 (44.6)	57 (48.7)
Deaths, n (%)	0	0
Patients with adverse events leading to drug discontinuation, n (%)	0	0
Patients with grade 3 or 4 adverse events, n (%)	2 (1.5)	2 (1.7)
Patients with serious adverse events, n (%)	1 (0.8)	1 (0.9)
eGFR <50 mL/min/1.73 m^2^	0	0
Adverse events, n (%)		
Upper respiratory tract infection	9 (6.9)	7 (6.0)
Nasopharyngitis	6 (4.6)	5 (4.3)
Headache	5 (3.8)	4 (3.4)
Fatigue	5 (3.8)	4 (3.4)
Nausea	5 (3.8)	4 (3.4)
Cough	4 (3.1)	3 (2.6)
Back pain	4 (3.1)	3 (2.6)
Dyspepsia	2 (1.5)	3 (2.6)
Diarrhea	2 (1.5)	0
Grade 3 or 4 laboratory abnormalities, n (%)		
Amylase >2 × ULN	2 (1.5)	2 (1.7)
Creatine kinase ≥10 × ULN	3 (2.3)	1 (0.9)
Absolute neutrophil count <750/ µL	1 (0.8)	0
Fasting LDL cholesterol >300 mg/dL	0	3 (2.6)
Fasting cholesterol >300 mg/dL	1 (0.8)	2 (1.7)
Fasting glucose >250 mg/dL	1 (0.8)	3 (2.6)
Non-fasting glucose >250 mg/dL	2 (1.5)	3 (2.6)
Urine erythrocytes	2 (1.5)	4 (3.4)
Urine glucose	0	2 (1.7)

### Safety

Both therapies were well tolerated. Most adverse events were mild-to-moderate in severity. There was no drug discontinuation due to adverse events during the follow-up period. A serious adverse event that occurred in one patient (0.6%) receiving ETV was ulcerative colitis and one patient (0.5%) receiving TAF was myoma of the uterus, which was deemed by the investigator not to be related to study treatments. No patient died during treatment. The most common adverse events were upper respiratory tract infection, fatigue, headache, nasopharyngitis, and nausea, according to patients’ reports. Our study found that skin itching occurred in three patients in the ETV group, but not in the TAF group. In contrast, gastrointestinal symptoms, such as nausea and dyspepsia, were more frequently reported in the TAF group than in the ETV group, as shown in Table 3.

## DISCUSSION

This 48-week extension study reinforces and expands upon the findings of the initial 24-week study, demonstrating the sustained superiority of switching from ETV to TAF in CHB patients with LLV. The prolonged follow-up provides crucial insights into the efficacy and safety of TAF in this specific patient population.

Our results confirmed the durable virological response was observed with switching from ETV to TAF. At week 48, the rate of CVR was significantly higher, and HBV DNA decline was more pronounced in the TAF group. Achieving CVR is crucial for long-term clinical benefits, making it the primary endpoint of our study.

Furthermore, a higher proportion of patients in the TAF group achieved ALT normalization at week 48 compared with the ETV group. This difference is likely attributable to the superior viral suppression observed in the TAF group. Although a faster ALT normalization, reflecting the quicker resolution of necroinflammation, may translate to faster liver fibrosis regression, a long-term follow-up is needed to confirm this.

We also compared qHBsAg level changes between the two groups. As qHBsAg is a key target for future HBV treatment, this comparison is significant. Although HBsAg loss was not observed in either group after 1 year of treatment, the median decline in serum qHBsAg was significantly greater in the TAF group. This difference may be attributed to higher serum interferon (IFN)-λ3 levels in patients receiving NAs, which can upregulate IFN-stimulated genes and inhibit HBsAg production in hepatoma cells (17–19).

In HBeAg-positive patients, TAF demonstrated a significantly higher rate of HBeAg loss and seroconversion at week 48 compared with ETV. However, these rates were lower than those observed in treatment-naive patients receiving TAF or ETV, potentially due to the lower baseline HBV DNA (253 IU/ mL) and ALT level (31 U/L) in our study population.

The superior virological efficacy of TAF, particularly in terms of HBV DNA suppression and HBeAg seroconversion, may be partly attributed to its distinct pharmacokinetic (PK) profile compared with ETV. TAF exhibits greater plasma stability, leading to the efficient delivery of TFV to hepatocytes and higher intracellular concentrations of its active form, tenofovir diphosphate (TFV-DP). This contrasts with ETV, which is rapidly absorbed and primarily eliminated by renal excretion. Although both are effective, TAF’s targeted delivery may contribute to its greater antiviral potency, particularly in patients with LLV on ETV (4, 9, 10).

Histological changes were not assessed in this study. Instead, we utilized LSM, APRI, and FIB-4, which are convenient, non-invasive, and quantitative measures of liver fibrosis. Previous studies have shown that LSM decline is more rapid in the first 6–12 months of ETV treatment, followed by a slower decline (20, 21). Given that our enrolled patients had already received antiviral therapy for at least 12 months, most had already achieved significant LSM reductions, making it challenging to observe further significant changes in our follow-up period. Our results showed no significant changes in LSM, APRI, or FIB-4 values in either the TAF or ETV group after 48 weeks of treatment. It is important to acknowledge that the relatively short 6- and 12-month repeat interval for non-invasive liver fibrosis tests (NITs), combined with the generally low baseline LSM values, may limit our ability to detect significant changes in liver fibrosis. Furthermore, liver inflammation, which may fluctuate during treatment, can influence LSM and laboratory-based scores like APRI and FIB-4. Therefore, these results should be interpreted with caution, and longer-term studies are needed to evaluate the impact of TAF on liver fibrosis.

Similar to ETV, over 1 year of treatment, TAF demonstrated small changes in renal function parameters, suggesting satisfactory renal safety compared with ETV. One strength of our study is that indicators of both glomerular functions (serum creatinine, eGFR) and renal tubule function (Uβ2-MG, UNAG, and urine microalbumin) remained unaffected after 48 weeks of treatment with either ETV or TAF. Our findings indicate that switching to TAF does not compromise renal safety compared with continuing ETV. However, a limitation of this renal analysis is that our patient population was relatively healthy in terms of renal function. To fully characterize the renal safety profile of TAF, longer-term studies and follow-up are necessary.

Concerning lipid safety, TDF treatment is known to have a “lipid-lowering effect” as previously described. According to clinical trials, patients who switched from TDF to TAF had greater increases in TC, LDL, and HDL cholesterol compared with those who continued TDF treatment due to high plasma tenofovir levels in TDF-treated patients, which has been linked to lipid reductions in patients on TDF (22, 23). According to previous reports, weight gain could be seen when receiving TAF in HBV-infected patients (24). To study the above findings, BMI and fasting lipid analysis were carried out in our current study. Over the course of 48 weeks, we found that both groups showed no significant differences in BMI. Regarding lipid profiles, the TAF group showed a more pronounced increase in TG and decrease in HDL compared with the ETV group; however, neither group exhibited significant changes from baseline. Moreover, there were no significant differences in TC, LDL cholesterol, or the TC/HDL ratio between the two groups. These results suggest a limited increase in cardiovascular risk. Thus, compared with TDF, TAF seems to demonstrate a ‘‘normal blood lipid level” effect. The clinical relevance of this lipid effect is unclear, and a precise mechanism for these changes has not been ascertained. We speculate the main reasons for lipid parameters changes include that (i) the chemical structure of TAF changes from the phosphate ester group (TDF) to the phosphate amide group; hence, it can be actively transported into hepatocytes, and (ii) TAF is mainly hydrolyzed by carboxylesterase 1, which is mainly expressed in the liver. Due to active transport energy consumption and large consumption of carboxylesterase 1 in the liver, the liver’s ability to metabolize lipids is decreased, resulting in dyslipidemia. The effects of antiviral drugs on the metabolism of patients should not be ignored. Blood lipid parameters and blood glucose are recommended for the follow-up of TAF treatment. Future studies need to focus on actual cardiovascular risk, not just lipid parameter changes.

Regarding safety, both treatment strategies were well-tolerated, with similar rates of adverse events, serious adverse events, and laboratory abnormalities. However, given that most CHB patients require lifelong therapy and often experience an increasing prevalence of comorbidities with age, careful monitoring and management of renal function and lipid parameters are crucial. Although TAF treatment led to modest increases in blood and urine glucose levels, these elevations were transient and primarily observed in patients with a history of diabetes mellitus or elevated baseline concentrations.

Some studies have focused on switching to TAF for CHB patients (17, 25, 26), we also carefully studied and summarized the advantages and disadvantages of our research. The strengths of our study include the following: (i) the analysis of our results was conducted using both IPTW and the raw cohort, which further reduces the potential for selection bias and confounding factors; and (ii) although it is known that switching to TAF can result in dyslipidemia in TDF-treated patients, we demonstrated that TAF seems to have a “normal blood lipid level” effect in ETV-treated patients, as deduced from a parallel comparison between continuing ETV and switching to TAF therapy. However, this study has several limitations: (i) despite employing IPTW to balance baseline characteristics, this study is still a real-world study, not a randomized controlled trial (RCT). The decision to switch to TAF or continue ETV was made by patients and their physicians, potentially introducing selection bias, as those who chose to switch might have had different underlying characteristics or expectations; (ii) although we used non-invasive methods like LSM, APRI, and FIB-4 to assess liver fibrosis, these methods provide indirect measures and cannot offer the precise histological evidence that a liver biopsy would provide; (iii) although our study included a larger sample size than many previous studies, it may still be insufficient to detect more subtle effects, and the 48-week observation period may not be long enough to assess hard clinical endpoints such as cirrhosis progression and HCC incidence; (iv) our study population primarily consisted of individuals from medical centers in China, which may limit the generalizability of our findings to other geographical regions and ethnicities, where genetic, lifestyle, and healthcare system differences could influence treatment outcomes; and (v) Uchida et al. (17) using multivariate analysis showed that the HBV genotype (B vs C) was a significant factor affecting treatment efficacy. Although genotypic analysis may provide more accurate clues to rescue treatments for LLV patients, we were unable to collect enough HBV genotype information due to the sensitivity of the genotyping technique (not all LLV patients have sufficient HBVDNA titers to be amplified for genotyping). Therefore, future studies with larger sample sizes and improved genotyping techniques are crucial to better understand the role of HBV genotype in LLV treatment outcomes.

In conclusion, switching to TAF for patients with LLV offers superior virological and biochemical benefits compared with continuing ETV, with similar safety profiles regarding renal function and lipid levels, ultimately leading to better long-term outcomes.

## Data Availability

This study is registered with the Chinese Clinical Trial Registry (ChiCTR, registration number: ChiCTR2400089257), and the full protocol, along with processed data supporting this study's findings, is publicly accessible at https://www.chictr.org.cn/showproj.html?proj=237926. Additional supporting data are available from the corresponding authors upon reasonable request.
